# A novel 40kDa N-terminal truncated carboxypeptidase E splice variant: cloning, cDNA sequence analysis and role in regulation of metastatic genes in human cancers

**DOI:** 10.18632/genesandcancer.193

**Published:** 2019

**Authors:** Xuyu Yang, Hong Lou, Ya-Ting Chen, Shui-Feng Huang, Y. Peng Loh

**Affiliations:** ^1^ Section on Cellular Neurobiology, Eunice Kennedy Shriver National Institute of Child Health and Human Development, National Institutes of Health, Bethesda, MD, USA; ^2^ Institute of Molecular and Genomic Medicine, National Health Research Institutes, Zhuna, Miaoli, Taiwan; ^3^ Department of Anatomical Pathology, Chung-Shan Medical University Hospital, Taichung, Taiwan

**Keywords:** hepatocellular carcinoma, HCC cells, ovarian cancer cells, glioma cells, MMP3

## Abstract

Carboxypeptidase E (CPE), a prohormone processing enzyme, is a 476- amino acid protein with a signal peptide in its N-terminus and is expressed in the nervous and the endocrine systems. Recent evidence indicate CPE plays various non-enzymatic roles in the endocrine and nervous systems and in various cancers. Besides wild type (WT) CPE, a 40-kDa CPE protein that localizes in the nucleus and cytoplasm has been described in embryonic mouse brain. In this study we have cloned this CPE variant encoding the 40kDa CPE-ΔN protein from human cancer cells. RACE assay and sequence analysis confirmed existence of this CPE variant mRNA, which has 198 nucleotides removed within the first exon and 589 nucleotides from the 3’-UTR, respectively, compared to WT-CPE mRNA. Bioinformatic analysis revealed that this CPE variant mRNA has a shortened open reading frame, which starts coding from the 3rd ATG relative to WT-CPE mRNA and encodes a 40kDa N-terminus truncated CPE protein. RT-PCR and Western blot analysis showed that 40kDa CPE-ΔN is expressed in multiple cancer cell lines and tumor tissues. Overexpression of this 40kDa CPE-ΔN variant up-regulated expression of multiple metastatic genes encompassing different signaling pathways, suggesting potentially an important role of CPE-ΔN in tumor metastasis.

## INTRODUCTION

Carboxypeptidase E (CPE), also known as carboxypeptidase H (CPH), is a prohormone processing enzyme that is encoded by the human *CPE* gene [1]. CPE is a 476- amino acid protein with a signal peptide in its N-terminus that is mainly expressed in brain and throughout the neuroendocrine system, including the endocrine pancreas, pituitary, and adrenal gland chromaffin cells [2]. In neuroendocrine cells, carboxypeptidase E is present in the secretory granules along with its peptide substrates and catalyzes the release of C-terminal arginine or lysine residues to generate biologically active peptides [3, 4, 5, 6].

Studies accumulated over the past several years suggest the complexity of CPE function, as reflected by its multiple domain structure. CPE contains several functional domains, a signal peptide directing the protein into the rough endoplasmic reticulum (RER) cisternae at the N-terminus, a catalytic domain in the middle, and a highly acidic C-terminal domain [7, 8]. Originally, CPE was characterized as an exopeptidase which is involved in the biosynthesis of peptide hormones and neuropeptides in the neuroendocrine system [9]. Recently, studies indicate CPE plays many non-enzymatic roles in the endocrine and nervous systems such as a sorting receptor, intracellular vesicle trafficking and localization of synaptic vesicles to the active zone, as well as neuroprotective effects on neurons [7, 10, 11, 12].

Aberrant expression of CPE has been found in several major tumors of epithelial origin, including lung, liver, colon, pancreatic and cervical cancers [13, 14, 15, 16, 17], as well as in neuroendocrine tumors such as insulinoma [3, 18], suggesting CPE might have a role in tumor progression. Evidences that CPE promotes tumor proliferation are emerging. Additionally, CPE treatment of hepatocellular carcinoma (HCC) MHCC97H cells during metabolic stress has been shown to mediate survival by up-regulating anti-apoptotic protein BCL-2 and other pro-survival genes through activation of the ERK1/2 pathway [19]. CPE may also have different effects on invasion and migration depending on the tumor type. In glioblastoma (GBM) cells, CPE has been identified as a regulator of RPS6 within the mTORc1 signaling pathway to reduce aerobic glycolysis and migration, which negatively affects tumor cell invasion and migration [20]; in contrast, CPE may promote migration and invasion in other tumor types such as cervical cancer and osteosarcoma [17, 21], although the underlying mechanism remains to be elucidated.

Changes in the RNA processing machinery result in multiple transcripts of a single gene, and consequently, proteins translated from the alternatively spliced mRNAs may differ from their amino acid sequence and biological functions. Recently, a N-terminal truncated 40kDa CPE, named 40kDa CPE-ΔN, has been identified from mouse embryonic mouse brain [22]. This CPE-ΔN variant acts in the nucleus to promote mouse embryonic cortical neuronal survival through up-regulation of expression of many genes that promote proliferation, differentiation, axonal guidance and cell migration, as well as programmed cell death, all events that take place during the formation of the nervous system [22]. Similarly, CPE has been found in the nucleus as revealed by immunohistochemical (IHC) staining in some human cancers [21, 23]. Despite previous studies implicating the CPE variant induces proliferation and invasion in various tumor cell lines and to promote growth and metastasis of tumors in orthotopic models of nude mice, and could potentially be a biomarker in predicting metastasis and recurrence of cancer [13, 16, 23], the complete characterization of this CPE variant has not yet been carried out. In order to better understand the structure, regulation and biological function of this CPE-ΔN variant, we have now undertaken to clone and characterize the mRNA. Here we carried out Northern blot and 5’/3’-RACE analysis on mRNA isolated from human HCC97H/L cells and found and characterized a CPE-ΔN mRNA encoding a 40kDa N-terminally truncated CPE variant. The effect of this 40kDa CPE-ΔN splice variant in inducing expression of metastasis-related genes was studied in HCC cells.

## RESULTS

### Northern blot analysis revealed a ~2.4kb and a ~1.7kb transcript of human CPE

**Figure 1:**
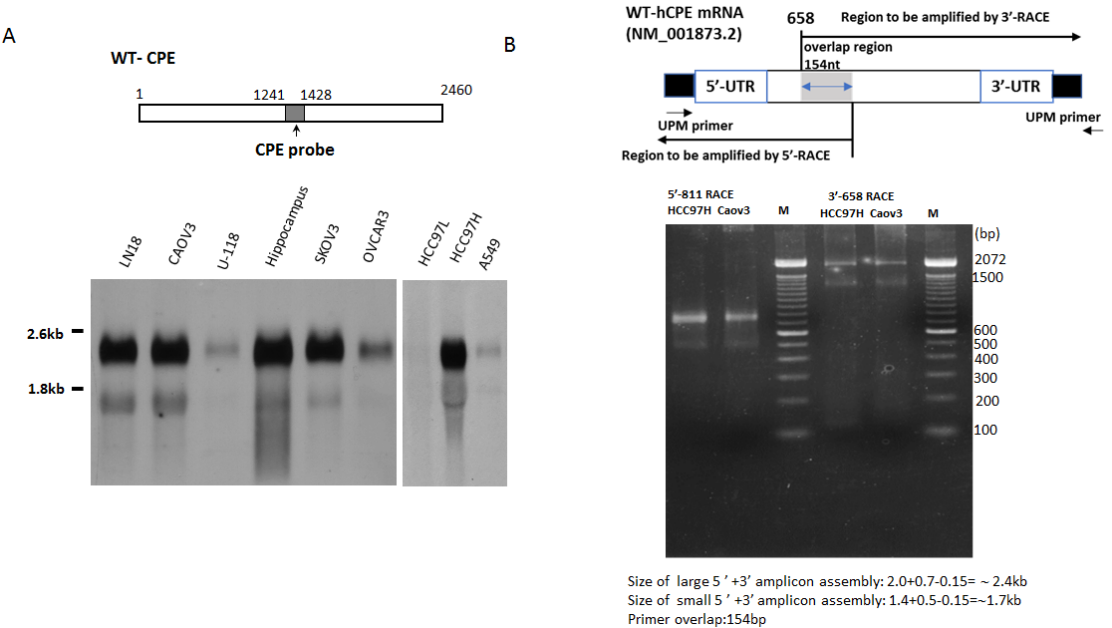
**A.** Upper panel: schematic representation of the DIG-labeled CPE probe (shaded box). The numbers refer to the probe covering the region in human CPE mRNA. Lower panel: Northern Blot, left to right, LN-18 mRNA, CAOV3 mRNA, U-118 mRNA, pooled human brain hippocampus Poly A+ mRNA from Clontech, positive control for WT-CPE, SKOV3 mRNA, OVCAR3 mRNA, HCC97L mRNA, HCC97H mRNA, A549 mRNA. **B.** Upper panel: schematic representation of the relationship of the primers used in the 5’/3’-RACE reactions to the CPE gene. The numbers refer to the positions in human CPE mRNA NM-001873.2. UPM primer binding sites were generated to the tail of both 5’-end and 3’-end first strand cDNA during the reverse transcription step. 5’/3’-RACE PCRs were performed by using primer set UPM/5’-811 and 3’-658/UPM, respectively; there is a 154 bp overlap region covered by CPE 5’-811 and CPE 3’-658 primers; Lower panel: 5’/3’-RACE reactions were performed with mRNAs from HCC97H, and CAOV3 cells to clone the CPE variant. Assembly of amplicons derived from 5’/3’-RACE PCR products indicates a 2.4 and a 1.7kb CPE transcript variant. M, 100bp DNA ladder. UPM primer provided by the Clontech 5’/3’ Smarter Race Kit.

In order to get an overall CPE expression profile and search for CPE transcript variant(s), Northern blotting analysis was performed with a panel of cancer cell lines including liver cancer lines HCC97H and HCC97L, ovarian cancer lines CAOV3, SKOV3 and OVCAR3, glioblastoma cancer lines LN18 and U-118 and lung cancer cell line A549, by using a DIG-labeled probe targeting CPE middle region from 1241-1428nt (Figure 1A). Of the eight cancer cell lines examined, LN-18, HCC97H, SKOV3, OVCAR3, CAOV3, HCC97H all expressed ~2.4kb and 1.7 kb transcripts of human CPE gene, while in the liver cancer line HCC97L, these transcripts were undetectable even with a large amount of mRNA input, possibly due to its very low abundance, since expression of CPE mRNA transcripts in those cells could be detected by a more sensitive RT-PCR assay [24]. Similarly, we observed that the liver cancer line, HCC97H, required a large amount of mRNA input compared with the other cancer cell lines for detection of both CPE transcripts, indicating a relative lower abundance of CPE transcripts within those cells. Of the two transcripts detected in all the various cancer lines studied, the upper one in the Northern blot appears to be ~2.4kb in size, which is approximately the same size as described by Lim et.al [1] and we identified this one as the WT-CPE transcript; the lower band, however, has never been described previously. The detection of more than one band for the CPE gene in the northern blot suggests the presence of multiple transcript variants. Additionally, the upper ~2.4kb band showed much stronger intensity than that of the lower one (~1.7kb), indicating lesser abundance of the new *CPE* transcript at the mRNA level compared to WT-CPE.

### 5’/3’/ RACE identifies the complete sequence of ~1.7kb CPE transcript

**Figure 2:**
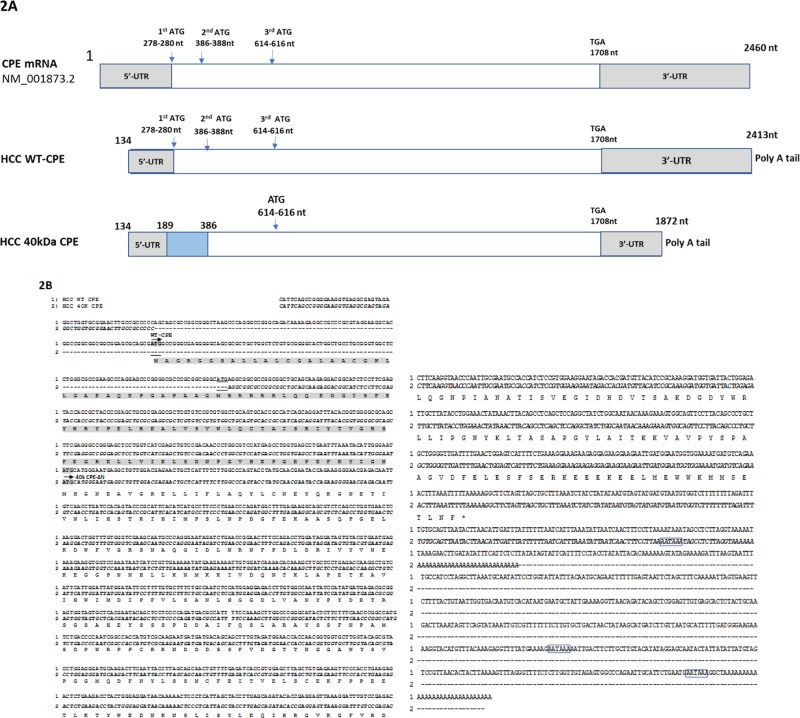
**A.** Schematic representation of the comparison of DNA and deduced amino acid sequences of homo sapiens CPE NM001873.2, wtCPE and its variant cloned from HCC cells; Numbers refer to the position in CPE NM001873.2. Both HCC wt CPE and the 40kDa CPE variant use an alternative transcription starting site at 134 nt, blue box in 40kDa CPE indicates an inner 198 bp deletion occurs in the exon 1, covering both partial 5’-UTR and coding region. **B.** Sequence comparison of DNA and deduced amino acid sequences of WT-CPE and its variant cloned from HCC cells; Both HCC WT-CPE and the 40kDa CPE variant use an alternative transcription starting site at 134 nt referring to the position in homo sapiens CPE (NM001873.2). Sequence 1, WT-CPE mRNA from HCC cells; Sequence 2 in italic bold, 40kDa CPE-ΔN mRNA from HCC cells. ATGs in shadow are the start codons for WT-CPE 40kDa CPE-ΔN, respectively. Dotted line in sequence 2 represents missing sequence of 189-386nt at the 5’-end and 1873-2413nt of 40kDa CPE-ΔN mRNA, respectively. Underlined ATGs are the start codons that were abolished by the deletion 189-386nt in 40kDa CPE-ΔN mRNA, which made the nearest downstream ATG at 614-616nt to be a putative translation initiation site and resides in-frame with human CPE. Amino acid sequence in shadow is the missing part of 40 kDa CPE-ΔN from WT-CPE. AATAAA, polyA tail signal. Arrows indicate translation starting sites for WT-CPE and 40kDa CPE-ΔN, respectively.

5’/3’ RACE were carried out separately in order to determine the nucleotide sequence of possible isoforms and splice variants of the CPE gene in human liver cancer cell HCC97H and ovarian cancer cell CAOV3. The detailed RACE priming strategy for cloning is shown in Figure 1B. There was a 154bp overlap between the PCR products. Specific 5’/3'RACE-PCR product were amplified by using primer 5’-811 and 3’-658, both of which showed dual bands on an agarose gel (Figure 1B). The RACE PCR products were purified for cloning and DNA sequencing. The cDNA sequence of a CPE variant, together with the deduced amino acid sequence detected in HCC97H cells, is shown in Figure 2A,B. Both WT-CPE and its variant from these cancer cells have a 133nt shorter 5’-UTR, as revealed by the sequence comparison to the corresponding sequence of human WT-CPE previously deposited in Genbank [NM_001873.2], indicating an alternative transcription initiation site was used; moreover, the 1.7kb CPE variant had a different 3’-UTR, which is 589 nucleotides shorter than the WT-CPE, possibly by using a polyA tail starting at position 1863nt after a polyadenylation signal of AATAAA. BLAST search revealed that there was a loss of 198 nucleotides from position 189-386 nt in the 1.7kb CPE transcript variant within exon 1. To confirm the 198 nt deletion occurs in the small CPE transcript variant, we carried out Northern blot with the probe targeting WT-CPE mRNA region 202-369nt; only the 2.4 kb CPE transcript was probed (Supplementary Figure 1). Since DNA mutation is often involved in alternative splicing in cancer cells [25], we extracted genomic DNA from these cancer cells and performed PCR with primer set 134/515 flanking the deletion region in CPE exon1. Deletion 189-386 was not found in these cancer cells (Supplementary Figure 2), which indicates the loss of 198 nucleotides within CPE exon 1 (189-386 nt) is not originally from DNA deletion.

ORF analysis showed the CPE variant encodes a N-terminal truncated form of CPE; due to the loss of 198bp in CPE exon 1, the first two ATG codons are removed, which renders the 3rd ATG to be a putative translation initiation site, and resides in-frame with the published sequence of human CPE (Figure 2A). The estimated molecular weight of this CPE variant is 40kD, which lacks the N-terminus 112 aa including the signal peptide, but the C-terminus enzymatic domain which is important for metallocarboxypeptidase activity remains intact.

### 40kDa CPE-ΔN transcript is expressed in various cancer cell lines and HCC tumors

**Figure 3:**
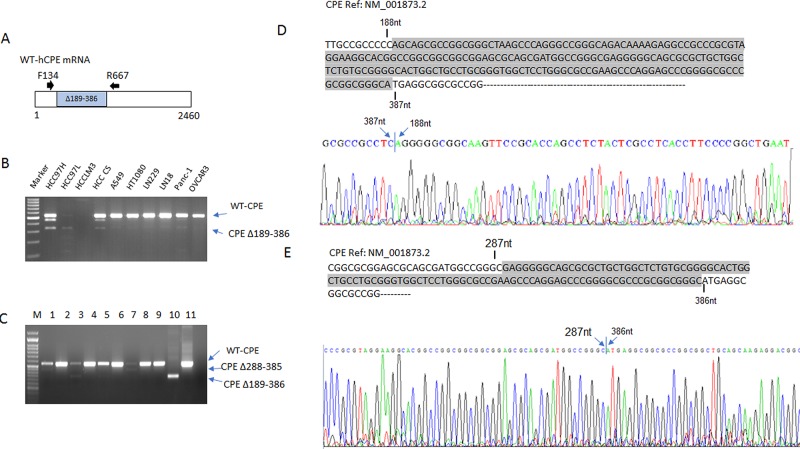
**A.** Schematic representation of the primer design to detect the CPE transcript variant. Due to the inner deletion in CPE exon1, primer set 134/667 generates a 534bp PCR product for WT-CPE, and a 336bp PCR product if there is Δ189-386 in CPE mRNA; **B., C.** RT-PCR was performed with cDNA derived from various cancer cell lines and liver cancer patients using primer set CPE F134/R667. Three CPE amplicons were amplified, upper band 534bp (wild type, upper arrow), middle band 436bp ;(Δ288-385, middle arrow), bottom band, 336bp (Δ189-386, bottom arrow). B: cell lines are indicated, HCC CS is a HCC patient tumor sample, C: HCC patients #1-9; 10, WT-CPE plasmid; 11, CPEΔ189-386 plasmid. Note the presence of all 3 amplicons in HCC97H cells and in HCC patient samples in varying amounts. **D., E.** The two small size PCR products were purified and subjected to DNA sequencing. Sequence alignment with WT-CPE (homo sapiens CPE NM001873.2) confirmed two deletion forms, Δ189-386 (upper) and Δ288-385(lower). Sequences in shaded area indicate deleted region in both forms.

To determine the expression of 40kDa CPE-ΔN transcript in various cancer cell lines and tumors, we performed RT-PCR with primer set (F134/R667) flanking the deletion region (189-386nt) based on the cDNA sequence of WT-CPE (Figure 3A). A 336 bp PCR product indicates the existence of the CPE variant due to the loss of 198bp in CPE exon 1 (Figure 3B). Sequence analysis (Figure 3D) verified that this splice variant exists in cancer lines and HCC patient tumors (see Figure 3B and 3C). Consistent with Northern Blot results, WT-CPE is more abundant than 40kDa CPE-ΔN transcript in all the cancer cell lines tested. Analysis of an additional 9 patient HCC tumors (Figure 3C) showed variability in the amount of the WT-CPE (534bp) versus the 40kDa CPE-ΔN (336 bp) PCR products. Furthermore, there is another smaller 436bp PCR product observed in these patient HCC tumors as well as in HCC97H cells (Figure 3B, lane 1). Sequence analysis (Figure 3E) of the 436bp PCR product from one of these HCC patient tumors indicate there might be another CPE splice variant with a deletion region at 288-385 nt which is identical to a previously deposited 2.1kb cDNA sequence (AK090962.1) in NCBI database, although we did not detect it in the RACE PCR.

### 40kDa CPE-ΔN protein localizes in cytoplasm and nucleus in liver and ovarian cancer cells

**Figure 4:**
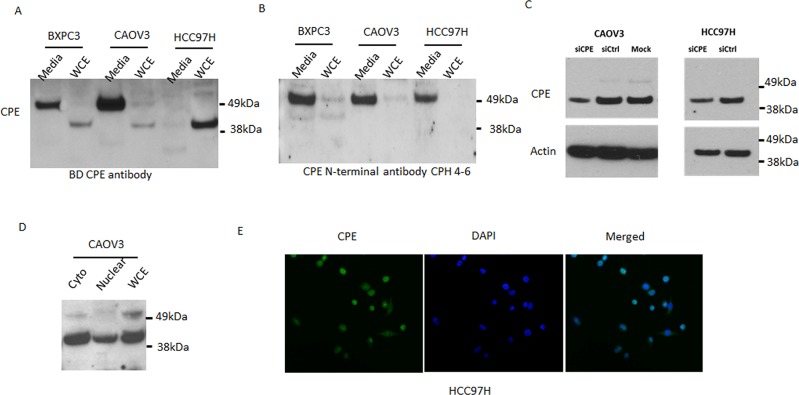
Expression of CPE and CPE-ΔN protein in various cancer cells Western blots were performed with two different antibodies, BD mouse CPE antibody and CPH 4-6 rabbit antibody, targeting CPE region at 49-200aa and the first N-terminal 1-18 aa, respectively. **A.** The BD CPE mouse antibody (against CPE aa 49-200) showed WT-CPE at 53kDa primarily in the cell media and little in whole cell extract (WCE), and CPE-ΔN variants at 40kDa in WCE; **B.** The N-terminal CPE antibody CPH4-6 (against the first 18 aa) revealed the 53kDa WT-CPE in the media and little to none in the whole cell lysate, but no 40kDa CPE- ΔN variants in the whole cell extracts; **C.** Both 53kDa WT-CPE and 40kDa CPE-ΔN variant were knocked down by siRNA against CPE as revealed by BD antibody; **D.** 40kDa CPE-ΔN was found in nuclear and cytoplasmic fractions in CAOV3 cells (BD antibody); **E.** Immunochemistry using BD mouse antibody showed CPE immunostaining in the nucleus of HCC97H liver cancer cells.

We examined expression of WT-CPE and its variant in liver and ovarian cell lines by Western blot (Figure 4). A 40kDa CPE-ΔN band was observed in cell lysates from HCC97H (liver) and COAV3 (ovarian) cancer cell lines using a BD anti-CPE antibody directed at amino acids 49-200 of CPE (Figure 4A). However, the 40kDa CPE-ΔN variant was not detected with an antibody (CPH 4-6) against the first 18 N-terminal amino acids of WT-CPE, whereas the WT-CPE protein present in the secretion medium was still detectable (Figure 4B), verifying the identification of this 40kDa band as the N-terminus truncated CPE protein. Treatment of the HCC97H cells and COAV3 cells with CPE siRNA suppressed expression of the 40kDa CPE-ΔN, further supporting its identity (Figure 4C).

Consistent with the nature of WT-CPE as a secretory protein, we detected large amounts of WT-CPE in the culture media in certain cell lines such as BXPC3 and CAOV3, but much less in HCC97H cells, whereas little to no WT-CPE were detectable in the cell lysates (Figure 4A, 4B), indicating the majority of WT-CPE were secreted outside cells via the secretory pathway. Unlike WT-CPE, the 40kDa CPE-ΔN was not secreted due to the lack of N-terminal signal peptide, instead, it was localized in both the cytoplasm and the nucleus (Figure 4D). Immunocytochemical studies of HCC97H cells which have virtually no detectable intracellular WT-CPE but only 40kDa CPE-ΔN (Figure 4A, 4B, 4C ctrl.) showed CPE-immunostaining in the nucleus, further supporting the localization of CPE-ΔN in the nuclear compartment (Figure 4E).

### Screening for metastasis-related genes up-regulated by 40kDa CPE-ΔN

**Figure 5:**
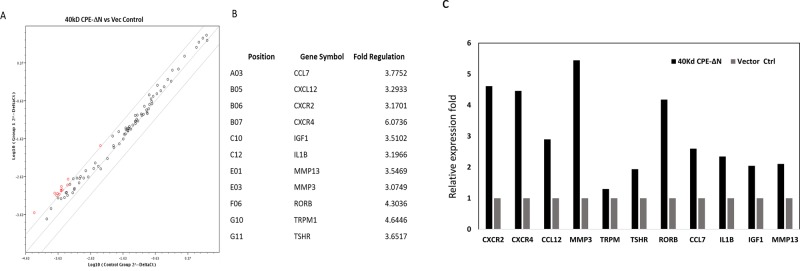
40kDa CPE-ΔN modulates expression of multiple metastasis-related genes Tumor Metastasis RT2 Profiler PCR Array was utilized to profile HCC97L cells transiently transfected with 40kDa CPE-ΔN or vector control for 48 hours. **A.** The corresponding scatterplot showed that 11 genes were upregulated (CCL7, CXCL12, CXCR2, CXCR4, IGF1, IL1B, MMP3, MMP13, RORB, TRPM1, TSHR) in 40kDa CPE-ΔN transfected HCC97L cells compared with vector control. The red circles indicate the selected genes with expression fold change larger than 2.0-fold. **B.** Tumor metastasis-related genes upregulated >3-fold by 40kDa CPE-ΔN; **C.** verification of CCXCR2, CXCR4, CCL12 expression in 40kDa CPE-ΔN transfected HCC97L by real-time RT-PCR.

Previous studies have shown that CPE-ΔN induces invasion of cancer cells [17, 26]. To explore the mechanism underlying the promotion of an aggressive phenotype induced by CPE-ΔN, metastasis-related genes that might be up-regulated between HCC97L cells transfected with 40kDa CPE-ΔN or vector control were screened using a human tumor metastasis PCR array. Among the 84 metastasis-related genes, 11 were upregulated (CCL7, CXCL12, CXCR2, CXCR4, IGF1, IL1B, MMP3, MMP13, RORB, TRPM1, TSHR) in transfected HCC97L cells (Figure 5A). CXCL12, CXCR2, CXCR4 and MMP3 were selected for further verification by real-time PCR using HCC97L cells transfected with the plasmid containing the 40kDa CPE-ΔN gene. The transcription of the CXCR2, CXCR4, CXCL12 and MMP3 genes were significantly increased by approximately 3-5 fold in the HCC97L cells transfected with the CPE-ΔN -expression plasmids, compared with cells transfected with mock plasmids (Figure 5A, B).

## DISCUSSION

Carboxypeptidase E (CPE) was first demonstrated as an enzyme that is involved with the biosynthesis of many peptide hormones and neuropeptides by removing C-terminal basic amino acids remaining after endoproteolytic processing [27, 28, 29]. Recent evidence indicate that WT-CPE plays a much broader role in the endocrine and nervous systems than previously recognized, as well as in various cancers [3, 10, 30, 31, 32]. In this study we have cloned the mRNA of a novel CPE variant and showed that it is ~1.7kb in size and encodes the 40kDa CPE-ΔN protein by Northern blot and 5’/3’-RACE. Analysis of the predicted amino acid (aa) sequence of this 40kDa CPE variant showed that it lacks the N-terminus 112 aa including the signal peptide, but the C-terminus enzymatic domain remains intact. By RT-PCR and Western Blot we showed that it is expressed in different cancer cell lines and clinical HCC samples. Overexpression of this 40kDa CPE-ΔN variant up-regulated expression of multiple metastatic genes encompassing different signaling pathways.

We detected both transcripts of WT-CPE (~2.4kb) and the variant (~1.7kb) in a panel of cancer cells by Northern blot, which differs from a previous report in which only a ~2.4kb CPE transcript was detected [1], possibly due to the low abundance of the small CPE variant since we were only able to capture it with large mRNA input. Consistent with the nature of WT-CPE as a secretory protein, the majority of it appears in the culture media, whereas the 40kDa CPE-ΔN can only be detected in the whole cell extracts as revealed by Western blot, since it lacks the N-terminal signal peptide required to direct it to the secretory pathway.

The loss of 198 nucleotides in CPE exon1 through intra-exonic splicing suggests the way 40kDa CPE-ΔN is generated in human cancer cells differs from its counterpart in the mouse, the latter uses a different transcription starting site to create the 40kDa CPE-ΔN and not through alternative splicing, and only appears to be expressed in embryos [22]. We did not find CPE Δ189-386 in genomic DNA isolated from these cancer cells (Supplementary Figure 2), indicating that the 40kDa CPE-ΔN mRNA is not directly transcribed from DNA. Further investigation is needed to determine whether this CPE splice variant is tumor specific, since many tumors have thousands of alternative splicing events not detectable in normal samples, which contribute to cancer development and progression [33, 34]. A recent comprehensive analysis of alternative splicing across multiple cancer types from 8,705 patients detected alternative splicing events and tumor variants by reanalyzing RNA and the whole-exome sequencing data; tumors have up to 30% more alternative splicing events than normal samples [33]. Alternatively, the splicing event may occur even in normal humans, since such a splice variant has been reported in human brain amygdala (Genbank accession #AK090962.1).

Our data suggest that 40kDa CPE-ΔN might play an important role in inducing invasion and metastasis by regulation of multiple metastasis-related genes. Function of CPE-ΔN in cancer cells is only just emerging, although studies have shown that CPE-WT promotes tumor cell proliferation in gliobastoma cells [35] and in PANC-1 cells (our unpublished data). In this study, we showed that a microarray analysis of CPE-ΔN transfected HCC97 L cells resulted in 11 metastatic genes being increased >2 fold. Of these genes, CXCL12, a chemokine and its cell surface receptor, CXCR4, and CXCR2 which are known to be involved in cancer cell proliferation and metastasis were verified by qRT-PCR to be significantly increased by ~3-fold in the HCC97L cells transfected with the CPE-ΔN expression plasmids. Evidence have shown that CXCR4 drives the metastatic phenotype in breast cancer through induction of CXCR2 and activation of MEK and PI3K pathways [36], while MMP3 contributes to the precision of epithelial cell branching via the processing of ECM components [37]. Thus identification of the mechanism underlying how CPE-ΔN up-regulates these genes could provide potentially new targets to interfere with progression of aggressive tumors.

In summary, in this study we have cloned a novel CPE variant, 40kDa CPE-ΔN, which lacks the N-terminal signal peptide, and has nuclear and cytoplasmic localization. Moreover, overexpression of 40kDa CPE-ΔN appears to be able to upregulate metastasis-related genes in HCC cells. Future investigations into the mechanism of action of CPE-N in this respect are necessary and could lead to identification of potentially useful targets for development of pharmaceutical agents against metastasis of tumors.

## MATERIALS AND METHODS

### Cell culture and plasmid transfection

The human HCC cell lines MHCC97H, MHCC97L, ovarian cancer CAOV3, SKOV3 and OVCAR3 cell lines, glioblastoma cell line LN-18, U118 and lung cancer line A549 were obtained from the American Type Culture Collection (Manassas, VA, USA). Cells were cultured in DMEM medium containing 10% (v/v) fetal calf serum at 37°C in a humidified 5% CO2. CPE-ΔN expressing plasmid was constructed by Genescript (Piscataway, NJ) and transfections were performed with Lipofectamine® 2000 (Invitrogen™; Thermo Fisher Scientific, Inc.) according to the manufacturer's instructions.

### HCC patient samples

Fresh frozen tumor tissues of 9 HCC patients were from Taiwan Liver Cancer Network (TLCN). This study protocol has been approved by the Institutional Review Board of National Health Research Institutes, Taiwan (NIRB EC0990701). Tumor from 1 HCC patient was from Maine Medical Center BioBank, Portland, Maine which operates under an Institutional Review Board (IRB) approved protocol and is overseen by the MMCRI Office of Research Compliance (FWA00003993).

### RNA isolation

Total RNA was isolated from different cell lines and HCC tumor samples by using TRIzol Reagent (Invitrogen) according to the manufacturer's instructions. Poly A+ mRNA was isolated by using NucleoTRAP mRNA kit from Clontech according to manufacturer's instruction.

### Northern blot analysis

A 188bp human CPE probe, covering middle region (1241-1428nt) of human CPE mRNA (GenBank accession number: NM_001873.2), was labeled with DIG (Roche DIG labelling kit, #11585550910) by PCR using primers hCPE F1241 and hCPE R1428 (see primer sequences in Table 1), pcDNA3.1-hCPE plasmid containing full length human WT-CPE cDNA was used as a template. PCR amplification was carried out in a 50 μl volume consisting of 30pg of WT-hCPE plasmid DNA, dNTPs (200 μM of dATP, dGTP, dCTP, 130 μM dTTP and 70 μM of DIG -11dUTP), 1 U of Taraka Hot Start DNA polymerase (Cat #R007A, Takara Bio USA) and 10 pmol of each primer. The amplification cycles involved an initial ‘hot start’ at 95°C for 5 min, followed by 31 cycles of amplification (94°C, 30 s; 56°C, 30 s; 72°C, 40 s) with a final extension step at 72°C for 5 min. Labeled CPE probe was purified by using QIAGEN QIAEX® II (Cat# 20051, Qiagen) kit as manufacturer indicated. Messenger RNA samples and DIG labeled RNA marker (Roche, #11526529910) were run on a denaturing formaldehyde gel and blotted to a nylon membrane using the Northern Max kit (Ambion, #AM1940). Membranes were hybridized overnight at 50°C. Following six washes (described in the Ambion protocol) the membranes were processed for immunodetection using an anti-DIG-AP antibody (1:10,000, Cat # 11093274910; Roche, Mannheim, Germany) and visualized by CSDP (1:100, Cat # 11655884001; Roche, Mannheim, Germany) against X-film at various time points as indicated by the manufacturer. A commercially available pooled brain hippocampus poly A+ RNA (Clontech Cat#636134) from 24 individuals isolated by two rounds of oligo (dT)-cellulose columns selection was used as a positive control for human CPE mRNA.

**Table 1:** Primers used in PCR

Human CXCR2 Forward	5’-CATGGCTTGATCAGCAAGGA-3’
Human CXCR2 Reverse	5’-TGGAAGTGTGCCCTGAAGAAG-3’
Human CXCR4 Forward	5’-CGTCAGTGAGGCAGATGAC-3’
Human CXCR4 Reverse	5’-TGCAATAGCAGGACAGGATG-3’
Human CXCL12 Forward	5-ATGCCCATGCCGATTCTTCG-3
Human CXCL12 Reverse	5-GCCGGGCTACAATCTGAAGG-3
Human GAPDH Forward	5’-CAACTACATGGTTTACATGTTC-3’
Human GAPDH Reverse	5’-GCCAGTGGACTCCACGAC-3’
Human MMP3 forward	5’-CCCTCTATGGACCTCCCCC-3’
Human MMP3 reverse	CATTCCACGCCTGAAGGAAGA
hCPE F1241	5'-GTA CCT GGA GGG ATG CAA GA-3'
hCPE R1428	5'-TGA AGG TCT CGG ACA AAT CC-3'
hCPE F134	5'-CAT TCA GCC GGG GAA GGT G-3'
hCPE R667	5'-GTA CTG GGC CAA GAA AAT GAGC-3'
hCPE 5’-811 (5’-RACE primer)	5'-GATTACGCCAAGCTTACCCACAAACCAGTCCTTGAGTTCACC-3'
hCPE 3’-658 (3’-RACE primer)	5’-GAT TAC GCC AAG CTT GGC CCA GTA CCT ATG CAA CGA ATA CCA-3’

### 5′/3’ –RACE

5/3′ -RACE was carried out using a Clontech Smarter RACE kit (Clontech, Palo Alto, CA, USA) according to manufacturer's protocol with modifications. Briefly, 5 μg of mRNA samples were heated at 70°C for 5 min and quickly cooled on ice before mixed with RT reaction mixture. Reverse transcription was carried out using SMARTScribe Reverse Transcriptase, a genetically modified MMLV in a 20 μl volume containing mRNA, oligo dT primer and Smarter II oligonucleotide A (5′ -RACE only) at 42°C for 2 hours. cDNA was diluted with 240 μl of Tricine-EDTA buffer and touch down PCR protocol was used to amplify the 5′ end of CPE transcript(s) using kit provided universal long primer, paired with CPE specific primer hCPE 5’-811 or 3’-658 (primer sequences available in Table 1). PCR amplification was carried out in a 50 μl volume consisting of 5 μl of diluted cDNA, 1 U of SeqAmp DNA polymerase (Clontech, Palo Alto, CA, USA), 1 μM of each primer. Touch down PCR was used to amplify 5’-end of CPE gene, cycles involve an initial ‘hot start’ at 95°C for 5min followed by 5 cycles of amplification 1 (94°C 30 sec 72°C 3 min), 5 cycles of amplification 2 (94°C 30 sec 70°C 30 sec 72°C 3 min) and 30 cycles of amplification 3 ( 94°C 30 sec, 68°C 30 sec, 72°C 3 min) with a final extension step of 72°C for 10 min. PCR products were analyzed on 1.8% agarose gels. Bands from 5’/3’-RACE were excised, purified and inserted into pRACE vector provided in the kit and sequenced with M13 primer.

### RT-PCR and real-time PCR

Two steps RT-PCR was performed to assess expression of CPE and its variant. Briefly, 2μg of RNA samples were heated at 70°C for 5 min and quickly cooled on ice before mixed with RT reaction mixture. Reverse transcription was carried out in a 20 μl volume containing mRNA, random primer, dNTPs and reverse transcriptase at 42°C for 90 minutes. cDNA was diluted with 180 μl of TE buffer and hot-start PCR protocol was used to amplify the CPE transcript(s) using primer set hCPE F134/hCPE R667 (primer sequences available in Table 1). PCR amplification was carried out in a 50 μl volume consisting of 5 μl of diluted cDNA, 1 U of SeqAmp DNA polymerase (Clontech, Palo Alto, CA, USA), 1 μM of each primer, cycles involve an initial ‘hot start’ at 95°C for 5min followed by 30 cycles of amplification (94°C 30 sec, 60°C 30 sec, 72°C 45 sec) with a final extension step of 72°C for 5 min. PCR products were analyzed on 1.8% agarose gels. PCR bands were excised, purified and sequenced. Real-time PCR reactions were performed using CyberGreen® PCR Master Mix (Applied Biosystems, Carlsbad, CA). The 2−ΔΔCt method was used to calculate the relative fold difference of mRNA expression.

### cDNA array analysis

The Human Tumor Metastasis PCR Array (SA Biosciences) was used, which includes cDNAs for 84 key representative genes involved in tumor metastasis. Briefly, total RNAs were extracted from CPE-ΔN and control transiently transfected HCC97L cells using the RNeasy Mini kit (Qiagen) according to the manufacturer's protocol. RNA quality was determined prior to gene expression analyses. For the cDNA synthesis, 5 μg of total RNA was reverse transcribed (RT) to first-strand cDNA according to the protocol provided by the RT2 Easy First Strand Kit (SA Biosciences). The resultant cDNA was diluted and mixed with the SA Biosciences RT2 qPCR Master Mix and water to a total volume of 2.7 ml. The reaction mixture (25 μl per well) was loaded into a 96 well plate for real-time PCR analyses performed on an Flex6 PCR machine following the instructions provided by the PCR array kit. The mRNA levels were normalized against housekeeping genes included in the PCR array (β-glucuronidase; hypoxanthine guanine phosphoribosyl transferase 1; heat shock protein 90 kDa α-glyceraldehyde-3-phosphate dehydrogenase and β-actin). Gene expression levels were calculated using the RT2 Profiler PCR Array Data Analysis Template v3.3 software and were expressed as folds of control cells. Unsupervised clustergram/heat map were generated by using the RT2 PCR array data analysis web portal (http://www.sabiosciences.com/pcrarraydataanalysis.php).

## SUPPLEMENTARY MATERIALS FIGURES AND TABLE


