# Supplementary material for: A novel 40kDa N-terminal truncated carboxypeptidase E splice variant: cloning, cDNA sequence analysis and role in regulation of metastatic genes in human cancers
Source: Genes Cancer. 2019;10(5-6):160–70. doi: 10.18632/genesandcancer.193 (PMC6872665; doi:10.18632/genesandcancer.193)
Supplement: Supplementary file 1 [file ganc-10-160-s001.pdf]

A novel 40kDa N-terminal truncated Carboxypeptidase E splice variant: Cloning, cDNA sequence analysis and role in regulation of metastatic genes in human cancers

Supplementary Materials

Genomic PCR

Genomic PCR was performed to detect CPEΔ189-386 within exon1. Briefly, genomic DNA from the cells was extracted using a DNAeasy kit from Qiagen according to the instruction. Hot-start PCR protocol was used to amplify the CPE exon1 using primer set hCPE F134/ hCPE R515 (primer sequences are as following: CPE F134: 5'-CAT TCA GCC GGG GAA GGT G-3'; CPE R515:5'- CCA CCG TGT AAA TCC TGC TGA-3')). PCR amplification was carried out in a 50 µl volume consisting of 100 ng of genomic DNA, 1 U of SeqAmp DNA polymerase (Clontech, Palo Alto, CA, USA), 1 µM of ach primer, cycles involve an initial 'hot start' at 95 °C for 5min followed by 30 cycles of amplification (94°C 30 sec, 60°C 30 sec, 72°C 45 sec) with a final extension step of 72 °C for 5 min. PCR products were analyzed on 1.8% agarose gels. CPEΔ189-386 and wild type plasmids were used as control.

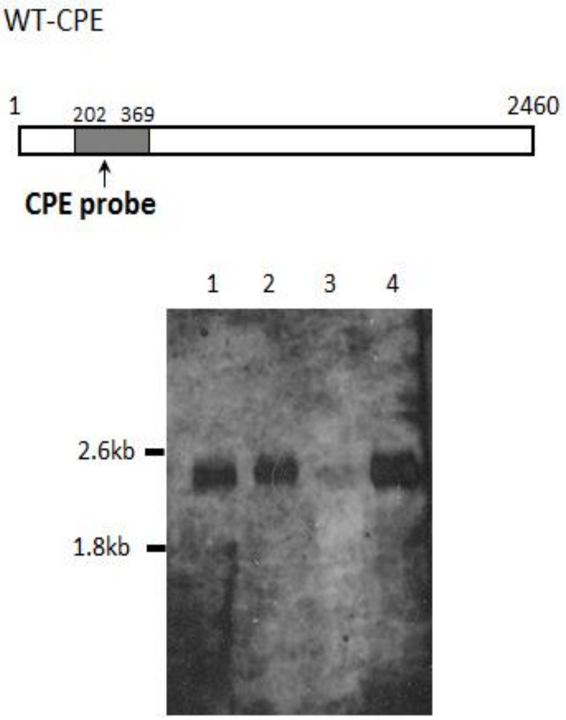

Supplementary Figure 1: Upper panel: schematic representation of the DIG-labeled CPE probe (shaded box) covering hCPE mRNA region 202-369nt. Numbers refer to the position of the probe in human CPE mRNA. Lower panel: Northern Blot, lane 1, LN18 mRNA; 2, CAOV3 mRNA; 3, U-118 mRNA; 4, pooled human hippocampus mRNA.

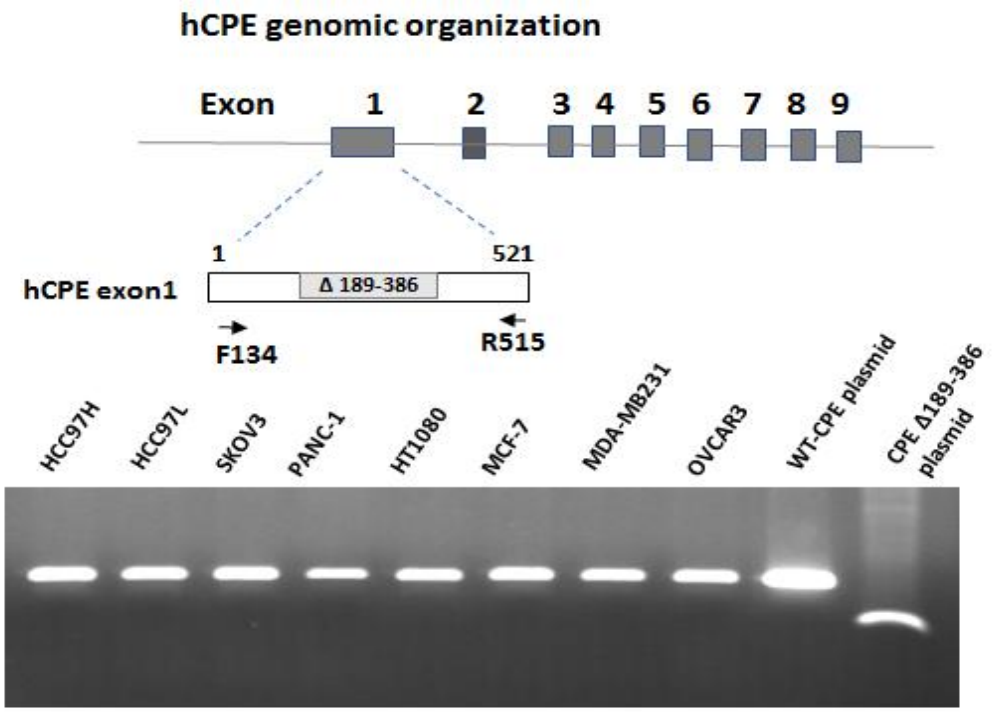

Supplementary Figure 2: Upper panel: schematic representation of the organization of human WT CPE gene. Primer set CPE F134/R515 was used for PCR to detect the CPEΔ 189-386 deletion within the 521bp long exon 1. Numbers of primer refer to the relative position to exon1. Lower panel: PCR was performed with genomic DNA derived from various cancer cell lines, a single PCR amplicon was amplified.

Supplementary Table 1: Primers used in PCR

Human CCL7 Forward	5’-ACAGAAGGACCACCAGTAGCCA-3’
Human CCL7 Reverse	5’-GGTGCTTCATAAAGTCCTGGACC-3’
Human IGF1 Forward	5’-CTCTTCAGTTCGTGTGTGGAGAC-3’
Human IGF1 Reverse	5’-CAGCCTCCTTAGATCACAGCTC-3’
Human TSHR Forward	5’-GAGTTTCCTTCACCTCACACGG-3’
Human TSHR Reverse	5’-CTGCTCTCATTACACATCAAGGAC-3’
Human TRPM1 Forward	5’-ACTACGTCATCCTGGTGCGGAT-3’
Human TRPM1 Reverse	5’-CTGGCTGAGTTTGCCTGGTTCT-3’
Human IL1B Forward	5’-CCACAGACCTTCCAGGAGAATG-3’
Human IL1B Reverse	5’-GTGCAGTTCAGTGATCGTACAGG-3’
Human RORB Forward	TGTGCCATCCAGATCACTCACG-3’
Human RORB Reverse	5’-GGTTGAAGGCACGGCACATTCT-3’
Human MMP13 Forward	5’-CCTTGATGCCATTACCAGTCTCC-3’
Human MMP13 Forward	5’-AAACAGCTCCGCATCAACCTGC-3’
Human CXCR2 Reverse	5’-CATGGCTTGATCAGCAAGGA-3’
Human CXCR2 Reverse	5’-TGGAAGTGTGCCCTGAAGAAG-3’
Human CXCR4 Forward	5’-CGTCAGTGAGGCAGATGAC-3’
Human CXCR4 Reverse	5’-TGCAATAGCAGGACAGGATG-3’
Human CXCL12 Forward	5-ATGCCCATGCCGATTCTTCG-3
Human CXCL12 Reverse	5-GCCGGGCTACAATCTGAAGG-3
Human GAPDH Forward	5’-CAACTACATGGTTTACATGTTC-3’
Human GAPDH Reverse	5’-GCCAGTGGACTCCACGAC-3’
Human MMP3 forward	5’-CCCTCTATGGACCTCCCCC-3’
Human MMP3 reverse	5’-CATTCCACGCCTGAAGGAAGA-3’
hCPE F1241	5'-GTA CCT GGA GGG ATG CAA GA-3'
